# Compartmental modeling for blood flow quantification from dynamic $^{15}$O-water PET images of humans: a systematic review

**DOI:** 10.1007/s12149-025-02014-x

**Published:** 2025-01-20

**Authors:** Oona Rainio, Riku Klén

**Affiliations:** Turku PET Centre, University of Turku and Turku University Hospital, Turku, Finland

**Keywords:** $^{15}$O-water positron emission tomography, Blood flow quantification, Compartmental modeling, Dynamic positron emission tomography

## Abstract

Dynamic positron emission tomography (PET) can be used to non-invasively estimate the blood flow of different organs via compartmental modeling. Out of different PET tracers, water labeled with the radioactive $^{15}$O isotope of oxygen (half-life of 2.04 min) is freely diffusable, and therefore, very well-suited for blood flow quantification. While the earlier $^{15}$O-water PET research has primarily focused on cerebral or myocardial blood flow quantification, the recent emergence of total-body PET scanners has enabled greater application possibilities for both PET imaging in general and also $^{15}$O-water PET based blood flow quantification in particular. However, to validate new methods, it is necessary to compare them to earlier research. To help in this process, we systematically review 53 articles quantifying blood flow via compartmental modeling. We introduce the articles organized within subcategories of cerebral, myocardial, renal, pulmonary, pancreatic, hepatic, muscle, and tumor blood flow and summarize their results so that they can easily be evaluated in terms of population characteristics of the patients such as age or sex ratio and their potential diagnoses. We compare how both the compartment model used and the potential corrections for arterial blood volume, non-perfusable tissue, spill-over from the heart cavities, and time delay caused while the tracer travels between different areas of interest are generally implemented in the articles. We also analyze the differences in the data pre-processing techniques. According to our results, the estimates of cerebral and tumor blood flow vary considerably more between the articles than those of myocardial blood flow. This might be caused by differences in the model approaches or the study populations. We also note that the choice of the unit for these estimates is quite inconsistent as certain researchers seem to prefer mL/min/g over mL/min/mL even if no weight or density parameter is present in the modeling. We encourage more research on sex- and age-based differences in blood flow estimates and organ-specific blood flow quantification studies for kidneys, lungs, liver, and other important organs besides brain and heart.

## Introduction

Positron emission tomography (PET) is a nuclear medicine imaging technique based on using short-lived radioactive tracers whose movements and distribution in the human body can be estimated due to the gamma radiation formed in the radioactive decay process [59]. Combined with magnetic resonance imaging (MRI) or computed tomography (CT) that shows the location of different anatomic structures, PET provides accurate information on the biology, metabolism, and function of the organs [2]. Water labeled with radioactive $^{15}$O-isotopes of oxygen (half-life: 2.04 min) is commonly utilized as a tracer in dynamic sequences of PET images to quantify cerebral, myocardial, or tumor perfusion [28].

Compartmental modeling is the standard method for analyzing dynamic PET images [2]. Namely, in a dynamic PET image, every voxel in the three-dimensional image has a time-activity curve (TAC) that shows the tracer concentration at that point with respect to different moments in time. By using a set of differential equalities, we can model the tracer exchange between an input function and specific TACs chosen from the image. Unlike several other tracers such as $^{18}$F-fluorodeoxyglucose ($^{18}$F-FDG), $^{15}$O-water is not metabolically inert and can therefore freely move between different tissues without accumulating them over time [28]. Consequently, a very simple model called one-tissue compartment model (1TCM) based on the principles of inert gas exchange as originally presented by Kety [26] in 1951 is still commonly used to describe the $^{15}$O-water delivery between the arterial blood and the tissue of interest.

However, while the basic 1TCM does not have many parameters itself, certain corrections might be necessary to account for the activity of the arterial blood within the tissue [31], the amount of non-perfusable tissue [39], or the delay caused the time it takes for the tracer to travel from one area of the human body to another [40]. Additionally, PET images can be corrected for radioactive decay, random coincides, dead time, scatter, and attenuation before computing the TACs. Furthermore, even if the same model is fitted to the data, the perfusion can be often estimated with both the first rate constant or the product of the second rate constant and the water partition coefficient. As different researchers estimate $^{15}$O-water perfusion with different choices of pre-processing techniques for PET images and modifications to the 1TCM, this raises the question about how comparable their results actually are.

To solve this issue, we wanted to systematically assess what models have been proposed for quantification of blood flow from $^{15}$O-water PET images and what kinds of estimates have been reported for humans. Given the fact that dynamic total-body PET imaging became very recently possible due to the emergence of the new-generation PET scanners with longer axial field of view [28], we did not want to limit our focus on specific organs. Instead, we wish to offer a comprehensive picture how the models designed for different organs and tissue types differ from each other and what perfusion values could be considered typical for baseline measurements at rest and during stress perfusion imaging, both for healthy humans and patients with different diseases.

In this review, we analyze 53 articles quantifying blood flow of humans with a compartment model from $^{15}$O-water PET images. First, we explain our methods of article selection and introduce the basics of compartmental modeling focusing on such models that appear in the chosen articles. We review the articles within subcategories defined by the type of perfusion. Most of the articles are related to cerebral blood flow (CBF), myocardial blood flow (MBF), or renal blood flow (RBF). We also briefly investigate pulmonary blood flow (PBF), pancreatic blood flow, hepatic blood flow, and muscle blood flow. Finally, we analyze several articles about tumor blood flow (TBF) related to various cancer diseases.

## Abbreviations

1TCM: One-tissue compartment model
ACZ: Acetazolamide
AIF: Arterial input function
AVF: Arterial blood volume fraction
CAD: Coronary artery disease
CBF: Cerebral blood flow
CT: Computed tomography
FDG: Fluorodeoxyglucose
GM: Gray matter
IDIF: Image-derived input function
MBF: Myocardial blood flow
MCA: Middle cerebral artery
MMD: Moyamoya disease
MRI: Magnetic resonance imaging
MS: Multiple sclerosis
NSCLC: Non-small cell lung cancer
PET: Positron emission tomography
PTF: Perfusable tissue fraction
PBF: Pulmonary blood flow
RBF: Renal blood flow
ROI: Region of interest
SOL: Steno-occlusive lesions
TAC: Time-activity curve
TBF: Tumor blood flow
VOI: Volume of interest
WM: White matter

## Article selection

**Fig. 1:**
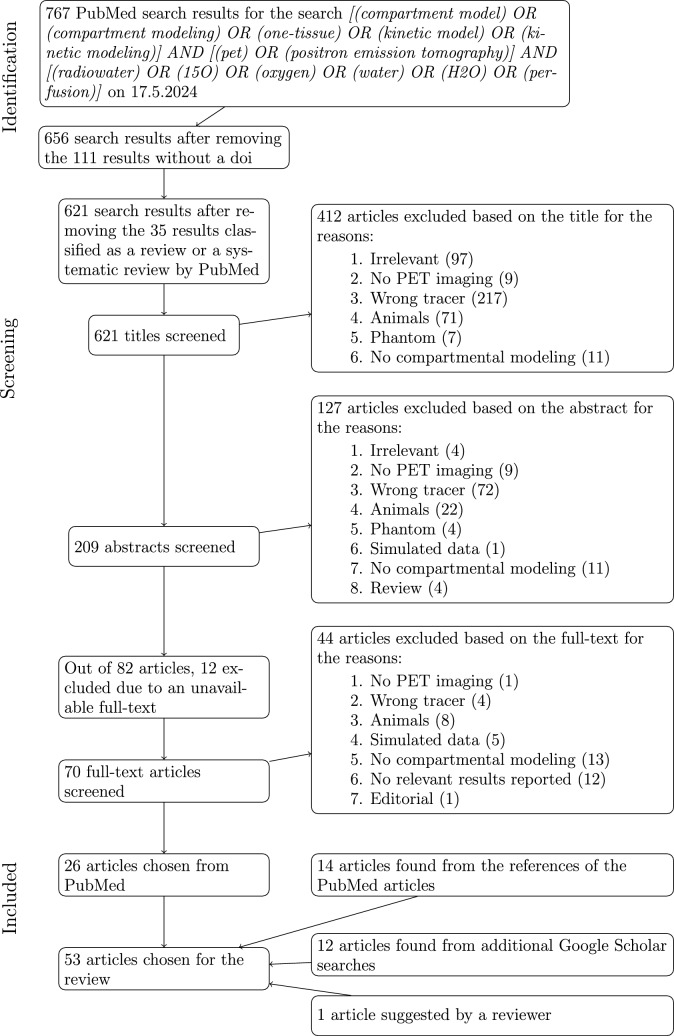
The article selection process of this review visualized as a flow diagram according to the PRISMA 2020 guidelines [45]

To select the articles, a PubMed search was first performed. Given that there are multiple important publications about compartmental modeling written already in the 1980s, the time period of the search was unlimited. We screened search results by their title, abstract, and full-text, leaving out such articles that were unrelated to PET imaging, used a tracer other than $^{15}$O-water, only contained data from animals, phantoms, or simulations instead of actual humans, or did not actually use any compartment model. After that, more articles were found from the references of the included PubMed articles. Additional Google Scholar searches were also performed to find articles about renal, pulmonary, pancreatic, hepatic, and muscle blood flow. The process of article selection is described in a more detailed way in the flowchart of Fig. 1.

## Modeling of dynamic PET data

### Pre-processing of PET data

Before kinetic modeling, several corrections can be done for PET data. First, to understand their importance, we must consider the process of PET imaging: A PET scanner estimates the tracer concentration in the human body by detecting the photons created in the annihilation of the positrons. A collision between one positron emitted from the unstable isotopes of the tracer and one electron of the body creates two photons, and the identification of one event requires that the PET camera detects both annihilation photons. Consequently, the PET data might include random coincidences caused by two unrelated photons mistaken as an event. However, events are also underestimated because some photons attenuate their energy before reaching the scanner while others might scatter around after colliding with a surface inside or outside of the body. PET scanners also require a minimum amount of time to process each event, so there is a possibility that some events might be lost if they occur during this dead time. Additionally, given the tracer concentration is measured based on its radioactivity, the radioactive decay must be taken into account, especially when the tracer is as short-lived as $^{15}$O-water. Because of this, the raw data given by the PET scanner is typically corrected for random coincides (referred as randoms), attenuation, scatter, dead time, and decay. Furthermore, different filtering methods can be used.

### Compartment models

The 1TCM requires two functions, $C_T(t)$ and $C_A(t)$, the former of which expresses the tracer concentration in the tissue with respect to time *t* and the latter of which shows the tracer concentration in the arterial blood. The actual model is fitted to the function $C_T(t)$, which is also the sole tissue compartment in the model, whereas $C_A(t)$ is the input function, generally referred as the arterial input function (AIF). The function $C_T(t)$ can be a TAC of a singular voxel in the dynamic PET image but, more commonly, it is the mean or the median TAC of several voxels in a specific region of interest (ROI) or a volume of interest (VOI) drawn in the image. The AIF can be computed by either measuring the activity in the blood through continuous arterial blood sampling during the imaging or, similarly to $C_T(t)$, as an image-derived input function (IDIF) of some ROI or VOI, typically placed in the ascending or descending aorta.

**Fig. 2:**
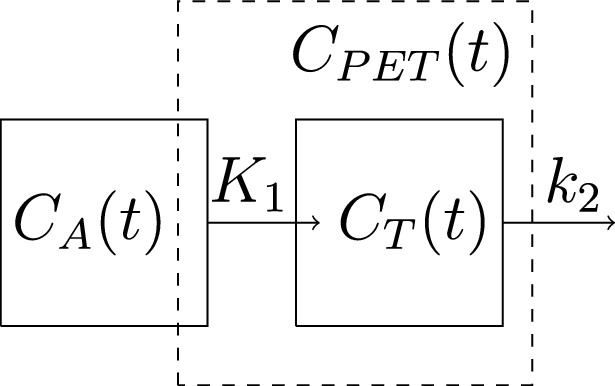
The 1TCM illustrated: Two compartments $C_A(t)$ and $C_T(t)$ so that the rate constant is $K_1$ for the tracer exchange from $C_A(t)$ to $C_T(t)$ and $k_2$ for the tracer departure from $C_T(t)$. The arrow of $k_2$ is sometimes drawn so that the tracer returns to $C_A(t)$ but, since $C_A(t)$ is an input function that is measured and not fitted by the model, no delivery from $k_2$ is actually added to its values. The compartment $C_A(t)$ is the tracer concentration in the arterial blood while $C_T(t)$ is the tracer concentration in the tissue. The measured tracer concentration of the tissue, $C_{\rm PET}(t)$, overestimates $C_T(t)$ due the concentration in the arteries within the tissue

Compartment models are based on the idea that a certain percentage of the contents of one compartment is always exchanged with another. In the 1TCM, we assume that $K_1\times 100\%$ of the arterial tracer concentration of the moment is delivered onto the tissue compartment under a time unit and, similarly, $k_2\times 100\%$ of the current tracer concentration within the tissue exits the tissue as shown in Fig. 2. Consequently, the change in the tracer concentration in the tissue can be described with the differential equation

1 $$\begin{aligned} \frac{\partial }{\partial t}C_T(t)=K_1C_A(t)-k_2C_T(t). \end{aligned}$$

Since we assume that the coefficients $K_1$ and $k_2$, known as rate constants, are constant with respect to time, this equation (1) can be solved as

2 $$\begin{aligned} C_T(t)=K_1C_A(t)\otimes e^{-k_2t}. \end{aligned}$$

Above, the sign $\otimes$ means the mathematical convolution defined as

$$\begin{aligned} f(t)\otimes g(t)=\int ^t_0f(u)g(t-u)\,\textrm{d}u. \end{aligned}$$

**Fig. 3:**
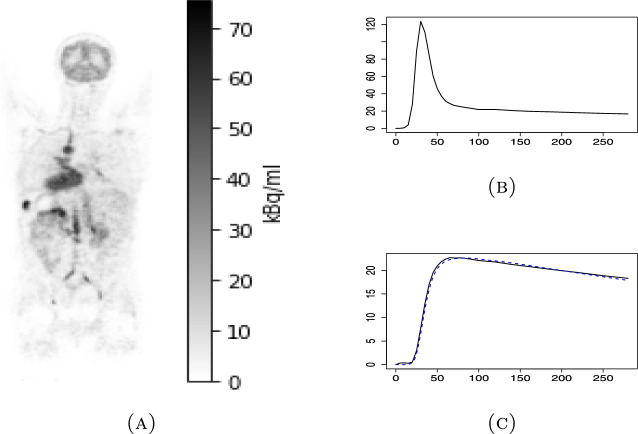
**A** One coronal plane in a dynamic total-body $^{15}$O-water PET image 50 s after the start of the imaging, **B** the AIF (unit: kBq/mL) of the same patient obtained as the IDIF of the aorta with respect of how many seconds has passed since the start of the imaging, and **C** the measured mean TAC (unit: kBq/mL) of the patient’s whole brain (in solid black line) and the TAC of the fitted 1TCM (2) with $K_1$=0.552 mL/min/mL and $k_2$=0.598 mL/min/mL (in dashed blue line)

An example of the TACs of $C_A(t)$, $C_T(t)$, and the fitted model can be seen from Fig. 3.

**Fig. 4:**
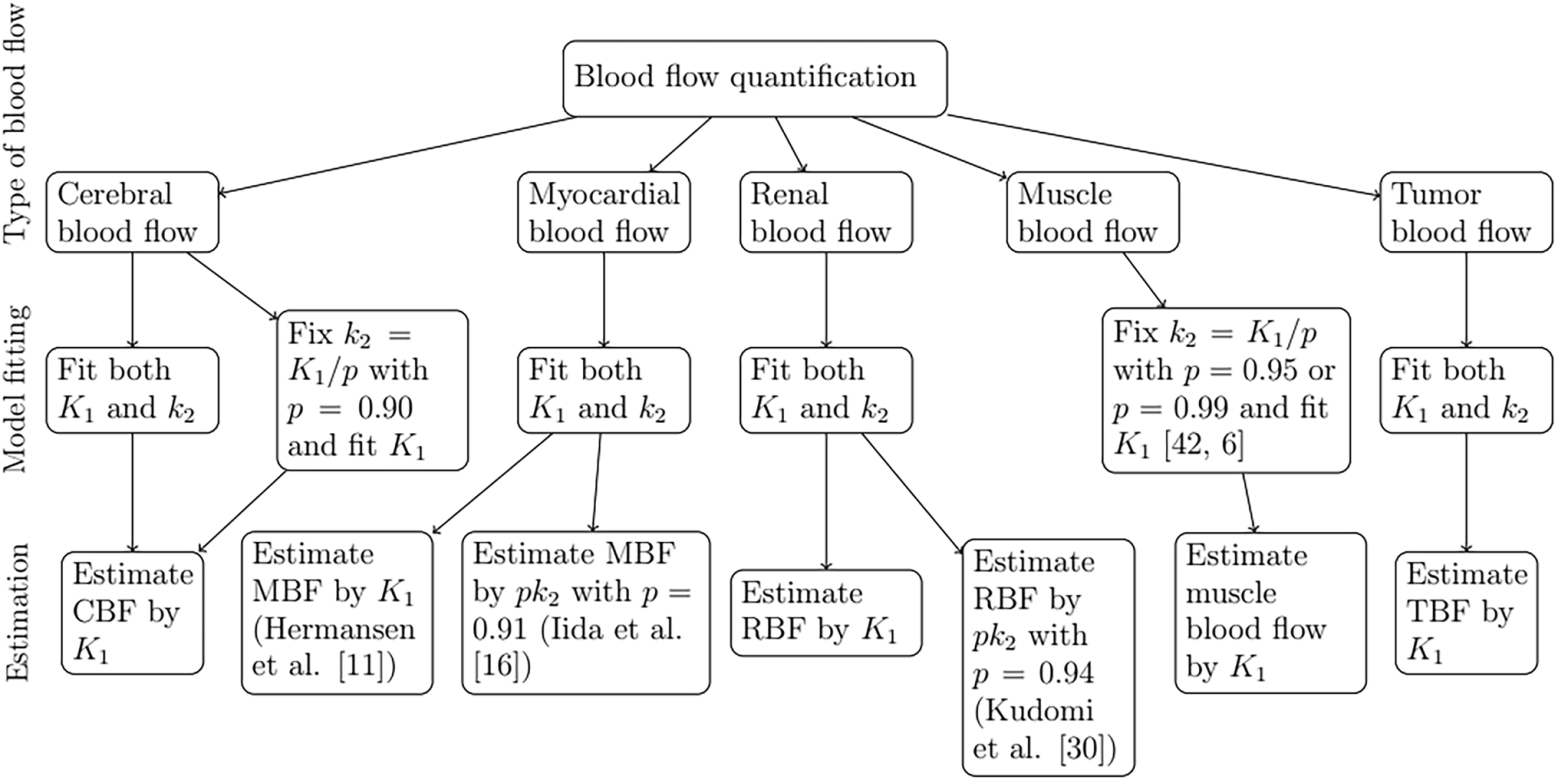
A flowchart summarizing which rate constants were fitted to the model and how the blood flow was estimated in the reviewed articles

The letter *F* is often used in (2) instead of the rate constant $K_1$, especially if the fitted rate constant $K_1$ is directly used to estimate the blood flow, while the rate constant $k_2$ can be replaced by *F*/*p* or $K_1/p$, where *p* is the water partition coefficient equal to the fraction $K_1/k_2$. Since neither of these choices typically affect the model fitting, we use the rate constants $K_1$ and $k_2$ in all the formulas here. However, it is sometimes possible that the value of the water partition coefficient *p* is fixed to a constant such as 0.90 [8, 9, 64], 0.95 [36, 42] or 0.99 [6]. In this case, we do not fit two independent rate constants to the model because this choice turns $k_2$ to a function of $K_1$. The possible options related to parameter fitting and blood flow estimation are summarized in Fig. 4.

To account for the activity measured from the tissue including also the activity caused by the tracer concentration in the arterial blood inside the tissue rather than just the actual within-tissue activity $C_T(t)$, we can define another function $C_{\rm PET}(t)$ comparable to the measured activity by using an additional parameter $V_A\in (0,1)$ for the arterial blood volume fraction (AVF) in the tissue so that

3 $$\begin{aligned} C_{\rm PET}(t)=(1-V_A)C_T(t)+V_AC_A(t). \end{aligned}$$

The 1TCM (1) with the AVF correction (3) as above can be written as

4 $$\begin{aligned} C_{\rm PET}(t)=(1-V_A)K_1C_A(t)\otimes e^{-k_2t}+V_AC_A(t). \end{aligned}$$

Alternatively, Ohta et al. [43] introduced a two-compartment model with separate parameters for transport and vascular distribution of brain water. Its solution can be written as

5 $$\begin{aligned} C_{\rm PET}(t)=K_1C_A(t)\otimes e^{-k_2t}+V_0C_A(t), \end{aligned}$$

where the parameter $V_0$ expresses the vascular volume of the arterioles to arterial capillaries. Notably, while this approach is technically based on a model with two tissue compartments, the equation (5) greatly resembles a simplified version of the AVF-corrected 1TCM (4).

If a notable portion of the ROI or the VOI is non-perfusable tissue or vasculature, it might be beneficial to include the perfusable tissue fraction (PTF) to the model (4). This is commonly done by an additional parameter $\alpha$ for PTF so that

6 $$\begin{aligned} C_{\rm PET}(t)=\alpha K_1C_A(t)\otimes e^{-k_2t}+V_AC_A(t) \end{aligned}$$

or

7 $$\begin{aligned} C_{\rm PET}(t)=\alpha (1-V_A)K_1C_A(t)\otimes e^{-k_2t}+V_AC_A(t), \end{aligned}$$

where $$\alpha \in (0,1)$$.

In cardiac modeling, it should be taken into account that, due to the limited resolution of PET and cardiac motion, high tracer concentration in the blood within the heart cavities often causes overestimation of the tracer uptake in the myocardial muscle. To address this spill-over, Hermansen et al. [11] suggested the following four-parameter model for the MBF quantification:

8 $$\begin{aligned}&C_{\rm PET}(t)=\alpha K_1C_A(t)\otimes e^{-(K_1/p+\lambda )t}\nonumber \\&+V_{\rm LVC}C_{\rm LVC}(t)+V_{\rm RVC}C_{\rm RVC}(t), \end{aligned}$$

where the MBF is estimated with the rate constant $K_1$, $\alpha$ is the PTF, *p* is the partition coefficient, $\lambda$ is the decay constant of 0.388/min, $V_{\rm LVC}$ and $V_{\rm RVC}$ are the spill-over fractions of the left and the right ventricular cavities, and $C_{\rm LVC}(t)$ and $C_{\rm RVC}(t)$ are the tracer concentrations in the left and the right ventricular cavities. A slightly simpler version of this model would be

9 $$\begin{aligned} C_{\rm PET}(t)=\alpha K_1C_A(t)\otimes e^{-(K_1/p+\lambda )t}+V_{\rm RVC}C_{\rm RVC}(t), \end{aligned}$$

which only has the spill-over term for the right ventricular. Alternatively, another version of the model (9) with the AVF correction of (3) is

10 $$\begin{aligned}&C_{\rm PET}(t)=(1-V_A-V_{\rm RVC})K_1C_A(t)\otimes e^{-k_2t}\nonumber \\&+V_AC_A(t)+V_{\rm RVC}C_{\rm RVC}(t). \end{aligned}$$

Another factor that might affect the model fitting is the delay caused by the time it takes from the tracer to travel from the area where the AIF was measured to the tissue of interest. This time delay can be either directly corrected to the data or, as suggested by Meyer [40], we can write

11 $$\begin{aligned}&C_T(t)=K_1e^{-k_2(t+\Delta t)}\int ^{t+\Delta t}_{\Delta t}C_A(u)e^{k_2u}{\rm d}u\nonumber \\&=K_1\int ^t_0C_A(u+\Delta t)e^{-k_2(t-u)}{\rm d}u \end{aligned}$$

instead of (2). In this way, the AVF correction of (3) results in the model

12 $$\begin{aligned} C_T(t)=V_AC_A(t)+(1-V_A)K_1\int ^t_0C_A(u+\Delta t)e^{-k_2(t-u)}{\rm d}u. \end{aligned}$$

As the velocity fields differ both between different vessels of the human body and between the vessels and the catheter of the devices used for arterial blood sampling, there is dispersion in the measured activity curve of the arterial blood when compared to the actual tracer input injected from the bolus. To avoid this discrepancy between the real AIF $C_A(t)$ and the measured AIF *g*(*t*), Iida et al. [15] proposed using a function

$$\begin{aligned} g(t)=C_A(t)\otimes \frac{1}{\tau }e^{-t/\tau }, \end{aligned}$$

where $\tau$ is a dispersion constant. It follows from this that

13 $$\begin{aligned} C_A(t)=g(t)+\tau \frac{\partial }{\partial t}g(t). \end{aligned}$$

We can add the delay time to this by defining [60]

14 $$\begin{aligned} C_A(t)=g(t+\Delta t)+\tau \frac{\partial }{\partial t}g(t+\Delta t) \end{aligned}$$

and then compute the model parameters either from 1TCM (2) without the AVF correction or from the AVF-corrected 1TCM (4).

### Fitting the model

There are multiple possible ways to fit the model to the TACs measured from the dynamic PET data. Nearly any optimization method can be used, and the task is straight-forward enough that the choice of the optimization method is unlikely cause significant differences in the results. Perhaps for this reason, several of the reviewed articles did not specify the exact method used for model fitting. However, many articles mentioned the basis function and linear least-squares methods proposed by Boellaard et al. [4]. In addition to linear least-squares, Christensen et al. [6] used nonlinear least-squares optimization performed with an in-house software tool and Kenny et al. [25] the standard weighted non-linear least squares, for instance.

### Converting the fitted parameters into blood flow and their units

The rate constant $K_1$ expresses the tracer exchange from the AIF to the tissue TAC under some specific amount of time. In case of CBF and TBF, the blood flow is nearly always estimated by fitting some variation of the 1TCM (4) and then directly estimating the blood flow from the value of the fitted rate constant $K_1$. However, the fitted rate constant $k_2$ multiplied by a constant value of the water partition coefficient might be sometimes used to estimate MBF or RBF. Typically, the time unit used in the unit of the blood flow is either originally in minutes or it is converted to minutes. For instance, if the fitted rate constant $K_1$ has a value of 0.1/min, then an estimated 10% of the current arterial tracer concentration is delivered to the tissue of interest every minute (min). Trivially, we can write $K_1$=0.1/min=0.1 mL/min/mL=0.1 mL/min/cm$^3$ to stress that from 10% of the activity concentration in a single milliliter (mL) of arterial blood is delivered to 1 mL or 1 cubic centimeter (cm$^3$) of tissue under 1 min. Additionally, if we know the density of the tissue of interest or we estimate that 1 cm$^3$ is approximately 1 g (g), then we can convert the unit of $K_1$ to mL/min/g.

## Estimated perfusion in different organs

### Cerebral blood flow

**Table 1:** Summary of the CBF articles including the reference to the article, the number of patients (*N*), their diagnosis, their age in years (mean± standard deviation, range in parenthesis), the male–female sex ratio, the region of the brain used to compute the CBF, the baseline CBF, the post-ACZ CBF, the unit of the baseline CBF and the post-ACZ CBF, reference to the equation of the model used to compute the CBFs, and the publishing year of the article

References	*N*	Diagnosis	Age	M:F	Region	Baseline CBF	Post-ACZ	Unit	Eq	Year
[49]	3	Healthy	31 (26–37)	3:0	GM	0.433±0.060	-	mL/min/g	(2)	1993
					WM	0.225±0.026				
[43]	9	Healthy	”Young”	NA	Global	0.35±0.11	-	mL/min/g	(5)	1996
[27]	21	Hydrocephalus	69.9±8.9 (49–81)	11:10	Global	0.382±0.131	-	mL/min/mL	(2)	1998
[22]	7	Healthy	49.1 (22–61)	4:3	Frontal	0.47±0.10	-	mL/min/mL	(5)	2001
					cortex					
[3]	12	Stenosis	75.0±5.0 (58-81)	5:7	Global	0.384±0.011	0.478±0.016	mL/min/g	(2)	2006
[13]	12	Healthy	36.2±13.2	12:0	GM	0.35±0.05	-	mL/min/cm$^3$	NA	2012
					WM	0.25±0.04				
[10]	16	Healthy	(20-24)	9:7	Global	0.485±0.056	-	mL/min/g	(4)	2014
[8]	13	MMD	9.7±7.1 (1-23)	5:8	Global	0.3058±0.0756	-	mL/min/g	(2)	2014
[9]	11	Healthy	34.7±13.8	11:0	Global	0.31±0.05	-	mL/min/cm$^3$	(2)	2014
					GM	0.38±0.05				
					WM	0.27±0.05				
	20	Type 1 diabetes	36.1±9.6	20:0	Global	0.30±0.05				
					GM	0.37±0.05				
					WM	0.26±0.04				
[64]	10	Healthy	25±3 (21–31)	10:0	GM	0.518±0.077	-	mL/min/g	(12)	2014
					WM	0.174±0.031				
[1]	20	Healthy	20	0:20	GM	0.56±0.034	-	mL/min/g	(5)	2017
			65		GM	0.47±0.023				
	38		20	38:0	GM	0.48±0.017				
			65		GM	0.46±0.015				
[21]	19	SOLs	68.8±9.8	18:1	MCA	0.465±0.073	0.542±0.086	mL/min/g	(5)	2017
[62]	22	Healthy	27.4 (18–40)	22:0	Global	0.349±0.034	-	mL/min/g	(4)	2017
[44]	11	Healthy	43.9±10.9	9:2	Global	0.479±0.059	-	mL/min/g	(5)	2018
[7]	18	Epilepsy (8)/	40±12	8:10	GM	0.75±0.22	-	mL/min/g	(4)	2021
		healthy (10)								
[14]	24	SOLs	67±11 (37–84)	20:4	MCA	0.479±0.074	0.553±0.086	mL/min/g	(5)	2021
	18	MMD	33±16 (12–70)	7:11	MCA	0.518±0.085	0.576±0.100			
[31]	25	MS/healthy	40 (23–56)	10:15	GM	0.45	0.60	mL/min/g	(4)	2021

In the article [1], CBFs of 20 women and 38 men between 21 and 65 years were used to estimate sex-specific CBFs at the age of 20 and 65 years

Out of the 53 articles chosen for this review, 17 were related to the quantification of CBF. The characteristics of the patient populations, the estimated CBFs, and the models used in these articles are summarized in Table 1. A few articles had both baseline CBF and the CBF during acetazolamide (ACZ) challenge.

The most popular model was the two-compartment model (5) introduced by Ohta et al. [43], though both the standard 1TCM (2) and the AVF-corrected 1TCM (4) were common. While most researchers fitted two model parameters for both rate constants $K_1$ and $k_2$ or, alternatively, the perfusion *F* and the water partition coefficient *p*, three articles [8, 9, 64] used a fixed partition coefficient of 0.90. Several articles, including [31, 49, 62], specified that the CBF was directly estimated from the fitted rate constant $K_1$ and there were no mentions of any corrections for $K_1$ to obtain the CBF in any of the articles.

In the great majority of the articles, the AIF was obtained from continuous blood sampling during the imaging as opposed to the IDIF from the aorta or some other target. Given the brain and aorta cannot be imaged simultaneously with a short axial field of view, this is understandable. However, Okazawa et al. [44] compared different non-invasive ways to derive AIF for computing the global CBF, including using the IDIF of the internal carotid arteries.

Most of the articles were very succinct when describing the pre-processing of the PET images. Both Huisman et al. [13] and Vestergaard et al. [62] stated that their PET data was corrected for randoms, scatter, attenuation, decay, and dead time, and most of the other articles mention at least two or three of these corrections, too. Filtering methods were also specified in a few articles: Fahlström et al. [7] reconstructed their PET data with a 5 mm Gaussian post-filtering. Both Klinge et al. [27] and Igarashi et al. [14] used a Hanning filter, also known as a Hann filter.

While several articles mention using dispersion correction, only three specify the choice of the dispersion constant: Kuttner et al. [31] derived the AIF from continuous blood sampling with a dispersion correction as in (13) with a dispersion constant $\tau$ of 15 s. Ito et al. [22] obtained the AIF by continuous measurement of arterial whole blood radioactivity with a beta probe, and then performed a dispersion correction with a dispersion constant of 4 s. Similarly, Okazawa et al. [44] also used a dispersion constant of 4 s but for an AIF obtained as an IDIF of the internal carotid arteries.

Furthermore, only a few articles also specified the method of time delay correction. These articles include both Islam et al. [21] and Igarashi et al., who [14] used a pixel-by-pixel time delay correction. Additionally, in research by Kuttner et al. [31], all PET data was interpolated linearly to one second time framing, and a time delay correction was performed by switching the AIF to maximize the dot product between the AIF and the median IDIF of the ten highest-intensity voxels in the first time frame with over 25% of the maximum intensity.

The majority of the subjects in the articles were adult men, especially in articles aiming to quantify the baseline CBF of healthy volunteers. However, Aanerud et al. [1] focused on studying the sex-based CBF differences. According to their results, young women have higher gray matter (GM) CBF than young men on average (0.56 vs 0.48 mL/min/g), but the women’s CBF of decreases faster as they age, so the sex-based differences eventually even out in older humans.

While the age and sex-based differences are not very noticeable between different articles, the brain area clearly affects the CBF values. In particular, GM CBF is about twice as high as white matter (WM) CBF in the five articles [9, 13, 49, 64] where both GM and WM CBF have been computed. Naturally, estimates of global CBF fall often between the reported GM and WM CBF values.

Several articles were focused on a specific disease. For instance, Klinge et al. [27] studied whether measuring the CBF of patients with chronic hydrocephalus is a reliable indicator of who to select for ventriculoperitoneal shunting. They investigated 21 patients scheduled for ventriculoperitoneal shunting due to symptoms of chronic hydrocephalus, such as gait disturbance, mental deterioration, and urinary incontinence. While they observed statistically significant clinical improvement in the patients with lower CBF after seven months from the shunting, the average pre-treatment global CBF of the chronic hydrocephalus patients in Table 1 is relatively to the global CBFs of healthy volunteers reported by Ohta et al. [43] and by Vestergaard et al. [62].

Bisdas et al. [3] studied CBF of patients with stenotic carotid disease. They used data from 12 patients, all of which were symptomatic and had experienced either a single transient ischemic attack, recurrent episodes of transient ischemic attacks, or a minor stroke. The average baseline CBF does not differ much from global CBF values reported for healthy volunteers and, while the post-ACZ CBF is lower than the other post-ACZ CBF values in Table 1, the other values are from different brain regions, not global.

Islam et al. [21] computed CBF for 19 patients with unilateral arterial steno-occlusive lesions (SOLs) while Igarashi et al. [14] computed CBF from baseline and post-ACZ PET scans of 25 patients with unilateral SOLs and 18 patients with Moyamoya disease (MMD). Based on the similarities of the data and same authors in both [21] and [14], it is likely that the PET images of the 19 patients in the research [21] are a subset of the PET images of the patients with SOLs in this research [14]. In both articles, the CBFs of the middle cerebral artery (MCA) regions were computed by first choosing several circular ROIs from the cortical territories of each transaxial PET slice and then using the model (5) modified with three different weight parameters. Consequently, while the estimated CBFs are higher than in most of the other articles, it is difficult to say whether it is because of the model, the MCA ROIs, or the condition of the patients. Goetti et al. [8] obtained lower CBF for MMD patients but they studied a population consisting of mostly children.

Fahlström et al. [7], van Golen et al. [9], and Kuttner et al. [31] all included data from non-healthy patients in their research, even though this was not their focus: Fahlström et al. [7] and Van Golen et al. [9] compared CBFs measured from $^{15}$O-water PET and pseudo-continuous arterial spin labeling MRI with datasets including patients with focal epilepsy and type 1 diabetes, respectively. Kuttner et al. [31] studied machine learning based non-invasive AIF prediction for measuring CBF from baseline and post-ACZ PET scans of 25 human subjects, including both multiple sclerosis (MS) patients and healthy volunteers. Notably, the CBF reported by Fahlström et al. [7] was much higher than the other CBFs of Table 1.

### Myocardial blood flow

**Table 2:** Summary of the MBF articles including reference to the article, number of patients (*N*), their diagnosis, their age (mean± standard deviation, range in parenthesis), male–female sex ratio, baseline MCF at rest, MBF during adenosine- or dipyridamole-induced stress perfusion imaging, unit of the MBFs, reference to the equation of the model used to compute the MBFs, and publishing year of the article

References	*N*	Diagnosis	Age	M:F	Rest MBF	Stress MBF	Unit	Eq	Year
[16]	7	Healthy/suspected	NA	NA	0.95±0.09	-	mL/min/g	(2)	1988
		angina pectoris							
[18]	9	Healthy	28±5 (21–34)	9:0	0.85±0.13	-	mL/min/g	(2)	1991
	4	Myocardial infarction	62 (52–67)	2:2	1.16±0.53				
[17]	5	Healthy	41±14 (27–58)	4:1	0.85±0.13	-	mL/min/g	(2)	1995
[11]	15	Ischemia (11)/healthy (4)	>40	14:1	0.84±0.23	-	mL/min/mL	(8)	1998
[24]	21	Healthy	45±8	21:0	0.89±0.15	3.51±0.45	mL/min/g	(8)	1999
[63]	11	Healthy	27±8	11:0	1.22±0.16	5.13±0.74	mL/min/g	(8)	2003
[48]	18	Healthy	40.0±14.4	18:0	0.93±0.26	3.76±1.21	mL/min/g	(2)	2006
[33]	23	Cancer	62±16	NA	1.08±0.30	-	mL/min/g	(4)	2008
[41]	48	Suspected CAD	66±7	37:11	0.988±0.275	2.653±0.900	mL/min/g	NA	2009
[35]	25	Suspected CAD	61 (31–78)	13:12	0.96±0.42	2.16±0.84	mL/min/g	(9)	2010
[58]	20	Healthy	28.4±8.9	20:0	0.71±0.11	3.09±0.97	mL/min/g	NA	2015
[47]	13	Non-obstructive CAD	62.5±5	15:5	0.81±0.17	3.21±1.03	mL/min/mL	NA	2018
	7	Obstructive CAD			0.82±0.14	2.15±1.10			
[54]	34	CAD/suspected CAD	70±8	27:7	-	2.51±0.89	mL/min/g	NA	2021
[38]	27	Ischemia	67.1±10.3	26:1	0.89±0.24	1.93±0.60	mL/min/g	(6)	2024

In [47], the patients were divided into two groups based whether they had obstructive or non-obstructive CAD but the age and sex information was only given for the whole patient population

The articles about MBF are summarized in Table 2. Quantification of rest and stress MBFs by $^{15}$O-water PET can be used to detect stenosis in the arteries and therefore provides important information about patients with a suspected or a diagnosed coronary artery disease (CAD). The most popular methods to estimating the MBF in the reviewed articles are based on the research by Iida et al. [16] and Hermansen et al. [11].

Iida et al. [16] introduced a method for measuring the MBF in the late-1980s. They performed PET scans for seven healthy human subjects, three of which were suspected of angina pectoris due chest pain complaints but had normal coronary angiography. The AIF was measured continuously with a beta-ray detection and corrected by using a dispersion constant less than 1 s. The ROIs were plotted to the septum, the anterior wall, and the lateral wall of the myocardium. The 1TCM (2) was then fitted for each ROI and the MBF was estimated with the fitted rate constant $k_2$ multiplied by the water partition coefficient fixed to the constant value of $p=0.91$ mL/g. The MBF in Table 2 is an average of the three MBFs from different ROIs. The same method was also used in their later research [17, 18], resulting in similar rest MBF values.

Hermansen et al. [11] introduced a new four-parameter model (8) and, unlike Iida et al. [16], estimated the MBF directly with the fitted rate constant $K_1$. The same method was also utilized by Kaufmann et al. [24] and Wyss et al. [63], who also computed stress MBF by infusing adenosine to the patients during the imaging. The MBF values reported by Wyss et al. [63] are notably higher than in other research, but Wyss et al. [63] also had the youngest patient population.

Pärkkä et al. [48] compared $^{15}$O-water PET and contrast-enhanced MRI for measuring myocardial perfusion reserve. Rest and stress PET scans were performed for 18 healthy male volunteers by using dipyridamole infusion to create hyperemia. The AIF was obtained as the TAC of the left ventricle with corrections for the limited recovery and the spillover from the myocardial signals. The MBF was computed with the method introduced by Iida et al. [16].

Lodge et al. [33] focused on the quantification of the TBF but they also computed the MBF in the same research article. Rest PET scans were performed for 23 cancer patients and the resulting data was corrected for dead time, scatter, and randoms. The AIF was obtained as the IDIF from either the left atrium or the left ventricular cavity and the tissue TAC from the myocardium. Like Iida et al. [16], they estimated the MBF with $k_2$ multiplied by the constant $p=0.91$ mL/g, though they used the AVF-corrected 1TCM (4) instead of the simpler 1TCM (2).

Lubberink et al. [35] studied the accuracy of the MBF measurements obtained without attenuation-correction. They collected rest and adenosine-induced stress PET images from 25 patients. Patients were referred to a PET scan due to a suspected CAD but none of them had a known history of CAD and no abnormalities were found from the results of the imaging. The PET data was corrected for scanner normalization, dead time, decay, scatter, and randoms. The AIF was derived from a VOI in the ascending aorta and the tissue TAC from a VOI over the right ventricular cavity. The MBF was estimated by the rate constant $K_1$ of the model (9) modified.

Takafuji et al. [54] compared MBF computed from $^{15}$O-water PET and dynamic perfusion CT. Their data was collected from 34 patients who underwent adenosine-induced stress PET imaging due to a known or a suspected CAD. A 1TCM was fitted to the TACs from the American Heart Association 17-segment model and the MBF was computed as a function of $K_1$ using also the information of the MBF estimated from the perfusion CT.

Maruo et al. [38] compared several estimates related to MBF of ischemic patients between electro-cardiogram gated and non-gated $^{15}$O-water PET scans. They used the model (6) so that the water partition coefficient was fixed to the constant value of 0.91 mL/g. They noted that the use of the electro-cardiogram gating produced significantly higher values for both rest and stress MBF compared to the non-gated PET, though the gating also presented a significantly higher reproducibility. The values in Table 2 are those obtained with the electro-cardiogram gating in non-ischemic heart segments.

Several researchers also used dedicated software programs for quantification of MBF: Nesterov et al. [41] computed MBFs with Carimas (Turku PET Centre, Turku, Finland) [50], Tomiyama et al. [58] used an unspecified in-house software, and Papanastasiou et al. [47] compared PMOD Software (PMOD Technologies, Fällanden, Switzerland) and Carimas. In research by Tomiyama et al. [58], the MBF was computed with a 1TCM by using the ROIs of the left ventricular chamber and the myocardium, while Papanastasiou et al. [47] computed the MBF with the 1TCM in Carimas and PMOD from semi-automatically created myocardial contours. The values in Table 2 are the ones obtained with Carimas.

### Renal blood flow

**Table 3:** Summary of the RBF articles including reference to the article, number of patients (*N*), their diagnosis, their age (mean± standard deviation, range in parenthesis), male–female sex ratio, rest ad stress RBF, unit of the RBFs, reference to the equation of the model used to compute the RBF, and publishing year of the article

References	*N*	Diagnosis	Age	M:F	Baseline RBF	Stress RBF	Unit	Eq	Year
[20]	8	Healthy	(20–74)	7:1	1.71±0.61	-	mL/min/g	(11)	1989
[30]	6	Healthy	58±5	NA	3.11±1.48	-	mL/min/g	(4)	2009
[46]	8	Healthy	60 (48–75)	5:3	1.8±0.3	2.2±0.6	mL/min/g	(4)	2018
	9	Type 2 diabetes	(52–85)	NA	1.3±0.4	1.6±0.5			

The research articles about RBF quantification are summarized in Table 3. It is worth noting that human kidneys consist two functionally very different parts, the highly vascularized renal cortex and the fluid-filled medulla. The articles quantifying the renal blood flow focus on the cortex where the blood flow is notably higher than it would inside the kidney.

In 1989, Inaba et al. [20] studied quantification of renal blood flow. Their data consisted of $^{15}$O-water PET images of seven healthy men with a mean age of 42.0 years (range: 20–74 years) and one healthy 55-year-old woman. The PET data was corrected for decay and ROIs were placed on the parenchymal portion of the kidney to derive tissue TACs whereas the AIF was obtained from arterial blood collected during the imaging. The RBF was computed with the time-delay-corrected 1TCM as in (11) but the fitted rate constant $K_1$ was divided by the volume of the kidney.

A different method was used by Kudomi et al. [30], who proposed fitting the AVF-corrected 1TCM (4) and then estimating the RBF by the fitted rate constant $k_2$ multiplied by the water partition coefficient fixed to the constant value of $p=0.94$ mL/g. Namely, they believed that the $K_1$ values underestimate the RBF due to the partial volume effect. With this new method, Kudomi et al. [30] obtained a much higher RBF value for healthy volunteers than the one in the earlier research by Inaba et al. [20].

However, by using the same method as in [30], Päivärinta et al. [46] obtained baseline RBF for eight healthy volunteers similar to the result of Inaba et al. [20]. In the same research, RBF was also computed also for a few subgroups formed within 17 atherosclerotic renovascular disease with a mean age of 69 years (range: 52–85 years) and a male–female sex ration of 7:10, and the lowest baseline and stress RBF values were obtained for 9 patients with type 2 diabetes and stenosed kidneys.

### Pulmonary blood flow

**Table 4:** Summary of the pulmonary blood flow (PBF) articles including reference to the article, number of patients (*N*), their diagnosis, their age (mean± standard deviation, range in parenthesis), male–female sex ratio, PBF, unit of the PBF, reference to the equation of the model used to compute the PBF, and publishing year of the article

References	*N*	Diagnosis	Age	M:F	PBF	Unit	Eq	Year
[52]	15	Healthy	(20–47)	6:9	1.41±0.22	mL/min/mL	(2)	1995
	5	Cardiomyopathy	(41–71)	1:4	0.98±0.28			
[39]	9	Lung cancer	60.2 (42–73)	6:3	1.4±0.3	mL/min/cm$^3$	(2)	2017
					5.0±0.6		(7)	

The PBF research is summarized in Table 4. Schuster et al. [52] quantified PBF with a simple model but by using pulmonary circulation blood from a right ventricular cavity ROI as the input instead of the AIF. Matsunaga et al. [39] investigated the impact on PTF parameter by computing PBFs of nine lung cancer patients with models similar to (2) and (7), though they also used an input from the right ventricular cavity. According to their results, the use of the PTF parameter greatly affected the resulting PBF estimates (see Table 4).

### Pancreatic blood flow

**Table 5:** Summary of the pancreatic blood flow articles including reference to the article, number of patients (*N*), their diagnosis, their age (mean± standard deviation, range in parenthesis), male–female sex ratio, pancreatic blood flow, its unit, reference to the equation of the model used to compute the blood flow, and publishing year of the article

References	*N*	Diagnosis	Age	M:F	Blood flow	Unit	Eq	Year
[29]	7	No lesions	64.8±12.5	14:12	1.13±0.482	mL/min/mL	(2)	2009
	8	Benign lesions			0.875±0.327			
	11	Malignant lesions			0.596±0.408			
[12]	15	Healthy	45±12	0:15	1.0±0.5	mL/min/mL	(2)	2015
	27	Morbidly obese	41±9	0:27	0.75±0.35			
[5]	8	Healthy	26.7±1.3	4:4	1.7±0.1	mL/min/g	(2)	2016
	7	Type 1 diabetes	25.9±0.9	4:3	1.3±0.1			
[32]	8	Healthy under saline infusion/ ketone body	59±7	5:3	1.55±0.34/ 1.61±0.50	mL/min/g	(2)	2016

The research on pancreatic blood flow quantification is summarized in Table 5. There is little variation in the models: all the four articles used a standard 1TCM with an IDIF from the abdominal aorta. Komar et al. [29] studied patients with suspected pancreatic malignancy and noted that the lesions decreased the flow also in nontumoral part of the pancreas. Honka et al. [12] and Carlbom et al. [5] also noted that patients with obesity and type 1 diabetes had a decreased pancreatic blood flow. Lauritsen et al. [32] studied pancreatic blood flow of healthy subjects under saline infusion or ketone body but noted no significant differences between these experiments.

### Hepatic blood flow

In 1999, Taniguchi et al. [56] introduced a dual-input model to measure hepatic blood flow by estimating the time delay first from the spleen. They performed $^{15}$O-water PET scans for 88 patients with a mean age of 57.9 years (range: 29–79 years) and a male–female sex ratio of 48:40. All the patients had normal liver function. The AIF was obtained from arterial blood sampling during the imaging and ROIs were drawn for liver and spleen. The mean total hepatic blood flow was 1.208$-$1.293 mL/min/g, depending on the liver segment considered, and the mean splenic blood flow was 1.333 mL/min/g. Taniguchi et al. [55] had also researched hepatic blood flow in an earlier study [55], obtaining a total hepatic blood flow of 1.00 mL/min/g and 0.674 mL/min/g for patients with healthy and cirrhotic liver, respectively. Similar model was later used by Rijzewijk et al. [51], who obtained a mean hepatic blood flow of 0.850±0.047 mL/min/mL for 18 healthy human subjects and notably decreased blood flow for patients with type 2 diabetes.

### Muscle blood flow

**Table 6:** Summary of the muscle blood flow articles including reference to the article, number of patients (*N*), their diagnosis, their age (mean± standard deviation, range in parenthesis), male–female sex ratio, muscles studied, their baseline blood flow, unit of the blood flows, reference to the equation of the model used to compute the blood flow, and publishing year of the article

References	*N*	Diagnosis	Age	M:F	Muscles	Blood flow	Unit	Eq	Year
[42]	12	Healthy	25±1	12:0	Femoral	0.023±0.003	mL/min/g	(2)	1996
[36]	5	Healthy	33.6 (24–38)	5:0	Femoral	0.034±0.003	mL/min/g	(2)	1997
[6]	10	Healthy	53 (41–69)	7:3	Lower legs	0.0213	mL/min/mL	(5)	2024
					Feet	0.0103			

The reviewed articles quantifying the muscle blood flow are presented in Table 6. Both Nuutila et al. [42] and Malminiemi et al. [36] focused on the impact of insulin on $^{18}$F-FDG uptake but they also computed baseline blood flows in femoral muscles from $^{15}$O-water PET scans of healthy men. They did not fit both rate constants $K_1$ and $k_2$ for the 1TCM (2) but instead fixed the water partition coefficient to 0.95 mL/g, resulting the equality $k_2=K_1/0.95$ (unit: /min). Similarly, while Christensen et al. [6] used the model (5) in their study of the repeatability of the $^{15}$O-water PET for skeletal muscle perfusion in the lower legs and feet, they also did not fit the rate constant $k_2$ but used a fixed water partition coefficient instead, though with a value of 0.99, not 0.95. In all research of Table 6, the muscle blood flow was directly estimated with the fitted rate constant $K_1$. The different choices of water partition coefficients are explained by the differences between the femoral muscles and the muscles in lower legs and feet.

### Tumor blood flow

**Table 7:** Summary of the TBF articles including reference to the article, number of patients (*N*), their cancer type, their age (mean± standard deviation, range in parenthesis), male–female sex ratio, TBF, unit of the TBF, reference to the equation of the model used to compute the TBF, and publishing year of the article

References	*N*	Cancer	Age	M:F	TBF	Unit	Eq	Year
[19]	11	Prostate	72.1 (54–83)	11:0	0.294±0.078	mL/min/g	(11)	1992
[37]	37	Breast	NA	0:37	0.33	mL/min/g	(5)	2002
[33]	23	Various, mostly renal cell	62±16	NA	1.01±1.18	mL/min/g	(4)	2008
[61]	11	NSCLC	59±12	9:2	0.29±0.23	mL/min/cm$^3$	(10)	2010
[25]	7	NSCLC (5)/melanoma (2)	66.1 (57–81)	NA	0.15$-$0.68	mL/min/cm$^3$	(4)	2014
[34]	9	Colorectal	54.4 (36–68)	4:5	0.44±0.25	mL/min/mL	(7)	2015
[57]	9	Prostate	70±4 (65–76)	9:0	0.24±0.20	mL/min/mL	(11)	2018
[23]	6	NSCLC	61.8 (42–73)	4:2	0.27±0.14	mL/min/cm$^3$	(10)	2021
	6		58.7 (42–71)	5:1	0.42±0.42			

In [23], the patients were divided into two groups of 6 patients based on their treatment but the TBFs in this table are pre-treatment so there should no significant differences between them. The article [33] is both here and Table 2 since both MBF and TBF were estimated in the same study

The articles about TBF are summarized in Table 7. As can be seen, the TBF varies greatly but, on the other hand, the reviewed articles have a very heterogeneous collection of different cancers. While the model varies, all the articles in Table 7 use the rate constant $K_1$ as the estimate of the TBF.

Both Inaba [19] and Tolbod et al. [57] studied TBF in the primary tumor of the prostate cancer patients by fitting the 1TCM (11). Inaba [19] only estimated the TBF directly with the rate constant $K_1$, while Tolbod et al. [57] reported the values for both the rate constants and compared the TBFs estimated from them. Interestingly, Tolbod et al. [57] noted that $k_2$ was significantly higher than $K_1$ because, according to them, $k_2$ only reflects TBF in the perfused parts of the tumor and is not affected nearly at all by the partial volume effect. The values in Table 7 are the values of $K_1$ to be compliant with the earlier research by Inaba [19].

Lodge et al. [33] aimed to quantify the TBF from $^{15}$O-water PET scans were performed for 23 patients with renal cell, kidney, colon, sarcoma, mesothelioma, bladder, and esophageal cancer by using an IDIF to derive the AIF. They estimated the TBF from the 1TCM (4) but obtained quite low results despite placing circular ROIs on the metabolically most active parts of the tumors according to according the $^{18}$F-FDG PET scans also performed for the patients.

Mankoff et al. [37] compared [Tc-99 m]-sestamibi uptake to TBF measured from $^{15}$O-water PET. Their data consisted of 37 patients with locally advanced breast cancer and the PET scans were performed together with continuous arterial blood sampling. The PET data was reconstructed with a Hanning filter and, like in the research by Lodge et al. [33], circular ROIs were placed on the metabolically most active parts of the tumors according to $^{18}$F-FDG PET images that were not a part of the study otherwise. TBF was computed from the model equivalent to (5), though with the expression $K_1/p+\lambda$ with $\lambda =0.388$/min for $k_2$ as in (8).

Van der Veldt et al. [61] computed the TBF of 11 non-small cell lung cancer (NSCLC) patients. PET scans were performed, and the resulting data was reconstructed to resolution of 6.5 mm. The AIF was obtained as the IDIF of the aorta and corrected for delay and dispersion, and another IDIF provided the tracer concentration in the right ventricular cavity. The tumor VOIs were drawn to the images by an experienced nuclear physician. The TBF was estimated by fitting the 1TCM (10).

Kenny et al. [25] studied $^{18}$F-fluciclatide as a PET tracer in cancer imaging by comparing its uptake to $^{15}$O-water perfusion. Seven patients with either NSCLC or melanoma were imaged with both $^{18}$F-fluciclatide and $^{15}$O-water PET, and continuous arterial blood sampling was also performed during the $^{15}$O-water PET imaging. The VOIs were manually drawn to the tumors to extract TAC and the AVF-corrected 1TCM (4) was used to compute TBF.

Lubberink et al. [34] researched the impact of a certain selective inhibitor of platelet-derived growth factor receptors and a receptor antagonist to water-perfusable tissue fraction to colorectal cancer metastases. PET scans were performed for nine CRC patients. The PET data were reconstructed with a 4.3 mm Gaussian filter and corrected for normalization, dead time, randoms, scatter, and attenuation. The AIF was obtained as the IDIF from the ascending or descending aorta VOI and the VOIs were also drawn for lung and liver metastases. The TBF was estimated by the rate constant $K_1$ of the PTF-corrected 1TCM (7) but the distribution volume $V_T$ was fixed to 0.96 mL/cm$^3$ so that $k_2=K_1/V_T$. The TBF in Table 7 is the baseline TBF of all lesions before receptors.

Katayama et al. [23] compared TBF of NSCLC patients before and after chemotherapy with or without bevacizumab. 12 NSCLC patients were divided in two groups so that six patients received chemotherapy with bevacizumab and the other six without bevacizumab. The data from PET scans was reconstructed with a 6 mm Gaussian filter. The VOIs were drawn to the ascending aorta, the right ventricular cavity, and each tumor with reference to CT images. The TBF was from the 1TCM (10) and the TBFs in Table 7 are both pre-chemotherapy so there should no significant difference with the two groups.

## Discussion

We reviewed 53 articles applying compartmental modeling to quantify blood flow from $^{15}$O-water PET data. Most popular model overall was the standard 1TCM (2) with no additional corrections but the AVF-corrected version (4) and the model (5) with an additional vascular volume term introduced by Ohta et al. [43]. While the great majority of the articles used the 1TCM with possible corrections for AVF, PTF, spill-over from the heart cavities, and time delay, also one dual-input model was presented for hepatic blood quantification in research by Taniguchi et al. [56].

During the review process, it was noted that the units greatly varied, even when same models are used to estimate blood flow from specific organs. The most common unit for blood flow was mL/min/g but very few of the articles specified using any density value to convert the unit /min, mL/min/mL, or mL/min/cm$^3$ of the rate constants $K_1$ and $k_2$ into mL/min/g. While certain researchers such as Iida et al. [16] and Kudomi [30] estimated MBF or RBF by the rate constant $k_2$ multiplied by the coefficient *p* in unit mL/g, the use of the unit mL/min/g seems quite illogical for CBF and TBF that are directly estimated from the value of the rate coefficient $K_1$.

According to Snyder [53], the standard tissue density of both brains and heart is 1.04 g/mL so there would only be a relatively minor difference between the original value of $K_1$ in mL/min/mL and the blood flow value converted into mL/min/g. Still, if the authors use such a model that the correct interpretation of the results is perfusion in the unit mL/min/mL and they directly report their results in mL/min/g instead of mL/min/mL without dividing this density 1.04 g/mL, they obtain a 4% error when compared to the results correctly converted into mL/min/g. Additionally, even if this conversion from the unit mL/min/mL into mL/min/g is performed correctly with the reference density 1.04 g/mL, there is an unavoidable possibility that the given subject has a different tissue density than this reference value. Namely, while brain and heart tissue are relatively homogeneous, some diseases might affect their density. Naturally, TBF quantification also raises the question about how to decide the reference density value in a reliable way as the tumor tissue is very heterogeneous depending on the type of the cancer and the location of the tumor. All these issues could be avoided by presenting the original estimates with the unit mL/min/mL.

When consulted, a more experienced researcher acquainted with the early history of kinetic modeling suggested that the unit mL/min/g was originally used so that perfusion values from PET would be comparable to the results of the microsphere method. Namely, the microsphere method was already well-established during the 1980s and its standard measurement is in the form of flow per a gram of tissue. We see from Table 7 that only the TBF articles published before 2010 use the unit mL/min/g but, for CBF, there does not seem to be a similar trend with respect to time. Perhaps, it is difficult to researchers to replace the unit mL/min/g when it is so commonly used in the original publications introducing the methodology.

By reviewing the reported numerical values from Tables 1-7, it can be seen that there is little variation in MBF between different articles but CBF and TBF differ much more. Naturally, the cancer type affects TBF and brain region CBF but, for instance, average baseline GM CBF of adults varies between 0.35±0.11 [43] and 0.75±0.22 mL/min/g [7]. As most of the articles only report the estimated blood flow values as opposed to including a supplementary file with the values of both rate constants, the estimated water partition coefficient, and the fitted parameters for AVF, PTF, and time delay, it is difficult to assess whether their results are reasonable or not. For instance, an interesting topic of future study could be to estimate how much the use of a constant value for the partition coefficient *p* produces differences in the blood flow estimates for organs such as kidneys where a fixed value of the partition coefficient *p* has been used relatively commonly in the past research. However, as it is, the individual differences between articles are so great that very few conclusions can be drawn from the impact of different illnesses on blood flow.

While some differences in the results could be potentially explained by the choices related to pre-processing techniques, this is also challenging to systematically compare between articles. Some articles mention specific corrections to PET data, but little to no details of their implementation are generally given. Additionally, if an article does not mention for instance an attenuation correction, it does not necessarily mean this correction was not performed because the authors might just consider it too trivial to mention.

One important aspect is the increased scanner resolution since the 1980s. The oldest study [15] among the ones reviewed here used a HEADTOME-III scanner with resolution of 13.4$\times$25.0$\times$40.00 mm$^3$ whereas in the most recent study [38], Maruo et al. reconstructed the PET images with a voxel size of 4.0$\times$4.0$\times$4.0 mm$^3$. High resolution PET scanners designed for brain imaging might have an even better resolution. This increased resolution has enabled the development of new models and the increased utilization of IDIFs during the compartment modeling.

Another noteworthy factor is that especially the older articles quantifying baseline blood flow of healthy adults only use data from male volunteers. Given Aanerud et al. [1] noted that there are sex-based differences in CBF of young adults, this topic could be studied further for different organs. Additionally, very little research was found on children or teenagers.

## Conclusion

We systematically reviewed 53 articles about blood flow quantification based on dynamic $^{15}$O-PET imaging via compartmental modeling. The majority of the articles focused on either CBF, MBF, or TBF whereas there was relatively limited amount of research on other important organs such as kidneys, lungs, or liver. While almost all articles estimated the blood flow as the first rate coefficient of 1TCM, both the methods of data pre-processing and the corrections for AVF, PTF, spill-over, and time delay varied in the model. It was noted that, compared to MBF, the estimates of CBF and TBF vary more between the articles. However, it is challenging to estimate whether differences in the final results are caused by the different modeling approaches or, for instance, the population characteristics of the human subjects and their potential diseases. More research would be needed from the sex- and age-based differences in the blood flow in organs other than the brain. Furthermore, we recommend that the researchers choose more carefully the units of their blood flow estimates.

## Data Availability

An excel file summarizing the PubMed search results is available on a reasonable request from the corresponding author.
